# RNA-Seq Reveals Pathways Responsible for Meat Quality Characteristic Differences between Two Yunnan Indigenous Chicken Breeds and Commercial Broilers

**DOI:** 10.3390/foods13132008

**Published:** 2024-06-25

**Authors:** Yong Liu, Xia Zhang, Kun Wang, Qihua Li, Shixiong Yan, Hongmei Shi, Lixian Liu, Shuangmin Liang, Min Yang, Zhengchang Su, Changrong Ge, Junjing Jia, Zhiqiang Xu, Tengfei Dou

**Affiliations:** 1 Yunnan Rural Revitalization Education Institute, Yunnan Open University, Kunming 650101, China; lydzq05091025@163.com (Y.L.);; 2 College of Animal Science and Technology, Yunnan Agricultural University, Kunming 650201, China; xiazhang1425@163.com (X.Z.); wangkun@126.com (K.W.); kmliqihua05@sohu.com (Q.L.); gcrzal@126.com (C.G.); greedtyas@126.com (J.J.); 3 Key Laboratory of Buffalo Genetics, Breeding and Reproduction Technology, Guangxi Bufialo Research Institute, Chinese Academy of Agricultural Sciences, Nanning 530001, China; 4 School of Biological and Food Engineering, Lvliang University, Lvliang 033000, China; 5 Institute of Science and Technology, Chuxiong Normal University, Chuxiong 675000, China; 6 College of Food Science and Technology, Yunnan Agricultural University, Kunming 650201, China; 7 Department of Bioinformatics and Genomics, College of Computing and Informatics, the University of North Carolina at Charlotte, Charlotte, NC 28223, USA; zcsu@uncc.edu

**Keywords:** chicken, meat quality characteristics, RNA-Seq, differentially expressed genes, fat metabolism

## Abstract

Poultry is a source of meat that is in great demand in the world. The quality of meat is an imperative point for shoppers. To explore the genes controlling meat quality characteristics, the growth and meat quality traits and muscle transcriptome of two indigenous Yunnan chicken breeds, Wuding chickens (WDs) and Daweishan mini chickens (MCs), were compared with Cobb broilers (CBs). The growth and meat quality characteristics of these two indigenous breeds were found to differ from CB. In particular, the crude fat (CF), inosine monophosphate content, amino acid (AA), and total fatty acid (TFA) content of WDs were significantly higher than those of CBs and MCs. In addition, it was found that MC pectoralis had 420 differentially expressed genes (DEGs) relative to CBs, and WDs had 217 DEGs relative to CBs. Among them, 105 DEGs were shared. The results of 10 selected genes were also confirmed by qPCR. The differentially expressed genes were six enriched Kyoto Encyclopedia of Genes and Genomes (KEGG) biological pathways including lysosomes, phagosomes, PPAR signaling pathways, cell adhesion molecules, cytokine–cytokine receptor interaction, and phagosome sphingolipid metabolism. Interestingly, four genes (*LPL*, *GK*, *SCD*, and *FABP7*) in the PPAR signal pathway related to fatty acid (FA) metabolism were elevated in WD muscles, which may account for higher CF, inosine monophosphate content, and AA and FA contents, key factors affecting meat quality. This work laid the foundation for improving the meat quality of Yunnan indigenous chickens, especially WD. In future molecular breeding, the genes in this study can be used as molecular screening markers and applied to the molecular breeding of chicken quality characteristics.

## 1. Introduction

Decades of intensive genetic selection have led to changes in indigenous Chinese chicken breeds regarding growth rate, body weight, efficiency of energy utilization, and so on. However, in most cases, the improvement of growth performance and reproductive performance has always been pursued at the expense of indicators such as meat quality performance and immune performance [1]. In particular, broiler breeding mainly focuses on daily gain and body weight, while ignoring other aspects. This results in meat quality-related indicators not being within the normal range and ultimately causes the meat quality to fail to meet the standards [2]. This will bring challenges to the development of commercial chickens. The Wuding Chicken (WD) and the Daweishan Mini Chicken (MC) are native Yunnan chicken breeds that have not been subjected to centralized selection during their formation. The WD is distinguished by its massive stature, heavy weight, quick growth, and delicious meat. The MC is distinguished by its small size, low weight, and strong resilience to disease. It is favored by researchers. Both breeds are considered to be ideal animal models for studying a number of important phenotypes [3]. Earlier studies showed that faster growth rates and larger body sizes are positively correlated with the number of muscle development genes. Slower growth rates and smaller body sizes were inversely correlated with cellular metabolism [4]. Therefore, highly selected Cobb broilers (CBs) were chosen for the study along with the unselected WD and MC.

RNA sequencing (RNA-seq) can detect genes and discover transcripts with high sensitivity and a large detection range. At present, it has been widely used in basic research, clinical diagnosis, and drug development [5]. Simultaneously, RNA-Seq is also favored by most livestock researchers. At present, breakthroughs have been made in molecular genetics and feed nutrition [6]. Park et al. [7] profiled gene expression using RNA-seq in the kidneys of broiler chickens fed diets containing distinctive concentrations of Ca^2+^ and identified seven candidate genes related to diminished weight gain and found that hypertension was associated with reduced weight gain. Willson et al. [8] revealed differentially expressed genes between broiler and layer chickens and found that the FoxO signaling pathway could be an active driver of the development of layer breeds. Yi et al. [9] examined the effects of different ammonia levels on the fat content in broiler breast muscle using RNA-seq and showed that exposure to high concentrations of air ammonia resulted in the differential expression of numerous genes (*CD36*, *ASB2*, *ACSL1*, *PLIN2*, etc.) in the broiler breast muscle.

Currently, little is known about the meat quality characteristics of these two indigenous chicken breeds. To fill this gap, two Yunnan native chicken breeds and the commercial CB breed were analyzed for meat quality characteristics, and RNA-seq was used to analyze differentially expressed genes (DEGs) in the three chicken breeds and identify key candidate genes related to meat quality characteristics, with the aim of laying a foundation for the molecular breeding of native Yunnan chicken breeds.

## 2. Materials and Methods

### 2.1. Animal Experimentation Ethical Statement

All studies involving animals were conducted in accordance with the Regulations on the Administration of Laboratory Animal Affairs (Ministry of Science and Technology of China; revised June 2004). All procedures conducted with the chickens were approved by the Yunnan Agricultural University Animal Care and Use Committee (approval ID: YAUACU C01). Sample collection was performed in accordance with the “Guide for the Care and Use of Laboratory Animals of Yunnan Agricultural University”.

### 2.2. Chicken, Diet and Housin

This study was carried out at Yunnan Agricultural University (Kunming, China). WD, MC, and CB eggs were incubated in the teaching practice chicken farm of Yunnan Agricultural University. WD eggs came from Wuding, Yunnan, China. MC eggs came from Pingbian, Yunnan, China. CB eggs were a commodity generation produced by Kunming Zhengda Co., Ltd., Kunming, Yunnan, China. For the experiment, 450 1-day-old healthy female chickens (150 from each chicken breed) were selected and raised in the teaching practice chicken farm of Yunnan Agricultural University. The experimental diet was formulated according to the nutritional requirements of the Chinese broiler (NY/T33-2004) starting diet (Phase I: 18.5% crude protein (CP) and 12 MJ/kg metabolic energy (ME)) to 30 days of age. From 30 days of age onward, chickens were fed a regular diet (Phase II: 17% CP and 12.1 MJ/kg ME) for up to 84 days (Table 1). Immunization was carried out according to routine immunization procedures (Table 2). There was free access to feed and water referring to the management standards of Yunnan Agricultural University for daily management (Table 3). Fly and rodent control, cleaning, and disinfection work were regularly carried out.

### 2.3. Measurement of Growth Performance

Growth performance was measured at each time point (28, 56, and 84 days old). The weighing was carried out using an electronic scale (Shanghai Yizhan Weighing Instruments Co., Ltd., Shanghai, China, YZ 0.01 g–10 kg). On days 28, 56, and 84, the chickens were fasted for 12 h and weighed, and the amounts of feed given and leftovers were recorded for the calculation of mean body weight, mean daily weight gain, mean daily feed intake, and Feed/Body weight gain. Feed conversion efficiency (FCE) was calculated based on feed intake. FCE = feed intake/weight gain.

### 2.4. Slaughter Procedure and Sample Collecting

Feed and water were restricted for 16 h and 12 h before slaughter. The body weight of the chickens was measured at 1, 28, 56, and 84 days old in the morning. Thirty WDs, MCs, and CBs were selected. Chickens were slaughtered by cervical dislocation in accordance with the National Experimental Animal Slaughter Standard of China. After slaughter, the phenotypes related to muscle development were evaluated (Table 4). At 84 days of age, left pectoral and leg muscle tissue (100 mg) was rapidly collected in 2 mL sterile test tubes after slaughter and frozen in liquid nitrogen before being transferred to an ultra-low temperature refrigerator at −80 °C for subsequent gene expression analysis by RNA-seq and Real-time Quantitative Polymerase Chain Reaction (qPCR). The left breast muscle (pectoralis major muscle) was taken in the same position as the left leg muscle (gastrocnemius muscle). All specimens were taken from the central portion of the widest part of the leg muscle and the ventral surface of the breast muscle (facing the skin), excluding the most superficial 3 mm. These samples were cut into 3 pieces (0.5 cm × 0.5 cm × 1.0 cm in size). The breast and leg muscles were stored in lyophilized tubes in liquid nitrogen at −80 °C for subsequent section preparation. The remaining left pectoral muscle and leg muscle were removed from the fascia and stored in a Ziplock bag in a refrigerator at −20 °C for future use for the determination of muscle quality physical and chemical indexes, amino acid (AA), and fatty acid (FA).

### 2.5. Measurement of Muscle Physical Parameters

Breast and leg muscles (5 cm × 3 cm × 1 cm) were taken, and the muscle pH was measured 45 min and 24 h after slaughter using a portable pH meter (Hanna-HI9025, Milan, Italy) (meat samples were cooled at 0~4 °C for 24 h immediately after slaughter).

Breast and leg muscles (5 cm × 3 cm × 1 cm) were taken, and the muscle color (L*, a*, and b* values) was measured by a Minolta-CR200 colorimeter (Suzhou Yuhong Trading Co., Suzhou, China) using a light source D65 with a measuring diameter of 8–11 mm.

Breast and leg muscles were taken as square pieces of meat approximately 2.5 cm thick and 6–7 cm long in a cooking bag, heated in a water bath at 80 °C to a center temperature of 70 °C, removed, and cooled down to room temperature. Breast and leg muscles (1 cm × 1 cm × 1 cm) were taken, and muscle shear force was measured by a digital meat tenderness meter (C-LM25, Beijing Tianxiang Feiyu Instruments & Equipment Co., Ltd., Beijing, China).

Fresh samples were taken within 2 h after slaughter and weighed, placed between 12 layers of absorbent paper on the top and bottom, and then placed on the steel ring allowing expansion and compression instrument operating table (DRK113, Shandong Deshun Instrument Co., Ltd., Jinan, China). They were then pressurized to 343 N and continued to pressurize at constant pressure for 5 min, were then quickly withdrawn from the pressure to weigh, and the water loss rate was calculated, $water\;loss\;rate\left(\%\right)=\left(W1-W2\right)/W1\times 100\%$ (W1: weight before pressing; W2: weight after pressing). After three measurements were taken for each sample group, the average value was determined.

### 2.6. Measurement of Muscle Chemical Parameters

The breast and leg samples were pounded and placed in a freeze-drying machine (FD-1C-50, Beijing Boyikang Test Instrument Co. Ltd., Beijing, China) to a constant weight, then crushed with a multi-functional grinder (DC-1000A, Zhejiang Wuyi Dingzang Daily Metal Products Factory, Jinhua, China) for later use. CP was determined with reference to GB 5009.5-2010 Determination of Protein in Foods; crude ash was determined with reference to GB 5009.4-2010 Determination of Ash in Foods; CF was determined with reference to GB/T9695.7-2008 Determination of Fat in Meat and Meat Products; water was determined with reference to CB/T 9695.15-2008 Determination of Water in Meat and Meat Products [10]. Next, 0.5 g of muscle tissue homogenate was taken and stored at −20 °C. After freezing, the samples were treated with 20 mL of 3.5% perchloric acid. Standards were then made, and standard curves were plotted. The content of inosine monophosphate was measured using high-performance liquid chromatography (HPLC) (Agilent 1200, Agilent, Santa Clara, CA, USA) under chromatographic conditions with a 20 μL injection volume. The inosine content was calculated using the formula X=C×V/(m×1000), in which X denotes the content of inosine in the sample, C denotes the concentration of inosine in the sample extract (μg/mL), V denotes the total volume of the sample extract (mL), and m denotes the mass of the sample (g).

### 2.7. Measurement of AA

Ten grams of breast and leg samples were taken, set at 65 ± 5 °C, dried to a constant weight (difference between two times ≤0.5 g), and equilibrated at room temperature for 24 h. The resulting sample was crushed, defatted, hydrolyzed, concentrated, and filtered. Finally, the AA content was measured using an automated amino acid analyzer (S433D, Sykam, Munich, Germany). Proline content was measured at 440 nm, and other amino acid contents were measured at 570 nm.

### 2.8. Measurement of FA

FA was measured using GC-MS (Trace 1310-ISQ 7000, Thermo Fisher, Waltham, MA, USA). The injection volume was 1 µL, the split ratio was 8:1, the inlet temperature was 250 °C, the ion source temperature was 230 °C, the transmission line temperature was 250 °C, the quadrupole temperature was 150 °C, and the carrier gas flow rate was 0.63 mL/min.

### 2.9. Cryosectioning and Hematoxylin and Eosin Staining

The frozen section technique was used to produce breast and leg muscle sections in this experiment. Briefly, an appropriate amount of the optical cutting temperature compound (OCT) (Sakura Finetek, Tokyo, Japan) was first added to a 25 mm × 40 mm × 5 mm embedding cassette (Citotest, Nanjing, China), then the muscle sample was placed, and OCT continued to be added until the sample was completely covered. The embedded samples were frozen with liquid nitrogen and fixed in a frozen sectioning machine (−25 °C) (Leica Biosystems, Wetzlar, Germany). The samples were cut into 10 μm slices and transferred onto glass slides, then stored at −20 °C until histological staining.

Muscle fiber morphological traits were determined using hematoxylin and eosin (H&E) staining. All staining reagents were purchased from Solarbio (Solarbio Life Science, Beijing, China), and the following protocol was used: hematoxylin, 5 min; water washing, 10 min; 1% Acid Alcohol Differentiation Solution, 25 s; water washing, 15 min; Eosin, 3 min; water washing, 2 min. After staining, the slices were dehydrated and transparently treated before being sealed with neutral resin. Using the Nikon ECLIPSE TI-S image acquisition system (Nikon Corporation, Tokyo, Japan), the diameter, single cross-sectional area, and density of muscle fibers at 200× magnification were examined. The fiber diameter and cross-sectional area were calculated using an image analysis system (Image-Pro Plus, Media Cybernetics, Rockville, MD, USA), and density refers to the number of muscle fibers in 1 mm^2^. For each sample, 3 different points on 3 images containing approximately 300 muscle fibers were estimated.

### 2.10. RNA-Seq Library Preparation and Data Analysis

A total RNA extraction kit (Bao Bioengineering Co., Ltd., Dalian, China) was used to extract the total RNA from the samples. The TruSeq^®^ RNA Sample Preparation Kit (Ilumina, San Diago, CA, USA) was used to construct RNA-seq libraries. The insertion size of the libraries was analyzed using an Agilent 2100 Bioanalyzer. One hundred and fifty cycles of paired-end sequencing were performed using the Illumina HiSeq 2500 Gene Sequencing Analysis System. The quality of the reads was checked using FastQC (Version 0.11.5, https://github.com/s-andrews/FastQC, accessed on 17 November, 2020). Possible adaptors were trimmed using Galore (Version 0.4.4), and cleaned reads were mapped to the reference genome (Gallus_gallus-5.0) using HISAT2 (Version 2.0.4, https://github.com/infphilo/hisat2, accessed on 17 November, 2020). Genes annotated in the Gallus gallus-5.0.94.gtf file were used to count the reads of each gene in each sample. Differentially expressed gene analysis was performed using the software DEseq2 (Version 1.20.0, https://github.com/mikelove/DESeq2, accessed on 17 November, 2020) and R language (Version 3.6.1, https://www.r-project.org/, accessed on 17 November, 2020) and the auxiliary software platform RStusio (Version 1.1.463, https://www.rstudio.com/, accessed on 17 November). Among them, the expression matrix and the sample information matrix, as well as the difference comparison matrix, were constructed for the counts obtained through DEseq2, and then standardization and difference analysis were performed to obtain differentially expressed genes (DEGs). Based on the DEGs obtained, the expression abundance of DEGs in each individual can be clustered. Combined with R software (Version 3.6.1, https://www.r-project.org/, accessed on 17 November, 2020) for analysis, the following software packages were used to complete the visual analysis of related data: gplots (version 3.0.1.1), AnnotationDbi (version 1.42.1), and ClusterProfiler (version 3.8.1).

### 2.11. qPCR Verification

Ten candidate genes were selected for qPCR validation. After extracting total RNA from breast muscle and leg muscle tissue, the total RNA reverse transcriptase kit (Takara, Dalian, China) was used to synthesize cDNA. The qPCR ABI 7500 Fast qPCR System using SYBR premix Ex TaqTM II (Takara, Dalian, China) was used to perform qPCR. The 2^−∆∆CT^ method was used to determine relative expression, and *β-actin* was used as the internal control for normalization of the results. The primer information for the 10 genes used for qPCR validation is shown in Table 5.

### 2.12. Data and Statistical Analysis

Excel 2013 (Microsoft Office, Washington, DC, USA) was used to process data, SPSS 21.0 (SPSS, Chicago, IL, USA) was used for statistical analysis, and the results were expressed as (mean ± standard deviation). Duncan’s method was used for significant difference analysis. All data visualization was performed using GraphPad Prism (version 9.4, GraphPad Software, San Diego, CA, USA).

## 3. Results

### 3.1. Comparative Analysis of Growth Performance

**Figure 1:**
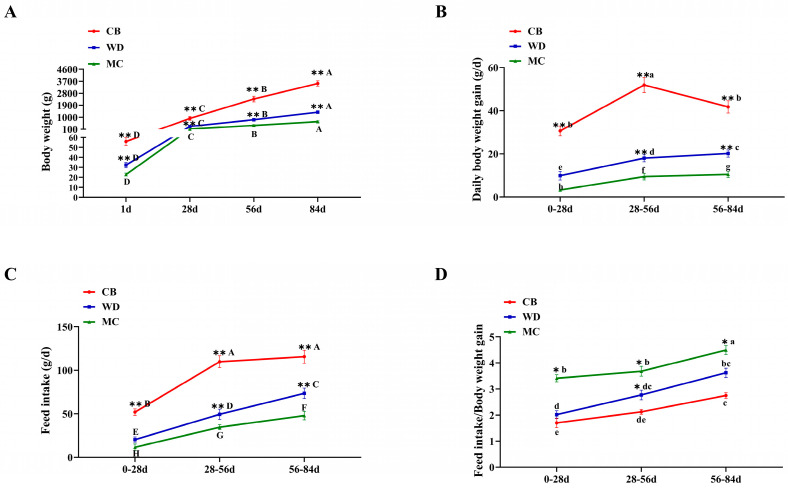
Growth performance of three chicken breeds. (**A**) Body weight. (**B**) Daily body weight gain. (**C**) Feed intake. (**D**) Feed/Body weight gain (feed conversion efficiency, FCE). Statistically significant differences in different breeds at the same age are indicated by * (*: *p* < 0.05; **: *p* < 0.01). Statistically significant different ages within a breed are indicated with letters (lower case letter: *p* < 0.05; upper case letters: *p* < 0.01).

The body weights of 1 d, 28 d, 56 d, and 84 d CBs and WDs were significantly higher than those of MCs (*p* < 0.01). One-day-old CBs, WDs, and MCs weighed 55.85 ± 3.26 g, 32.25 ± 2.63 g, and 22.75 ± 1.35 g, respectively, while 84 d CBs, WDs, and MCs weighed 3537.20 ± 172.91 g, 1378.40 ± 53.53 g, and 672.00 ± 56.18 g, respectively (Figure 1A). The daily weight gain and feed intake of 1–28 d, 28–56 d, and 56–84 d CBs and WDs were significantly higher than those of MCs (*p* < 0.01) (Figure 1B,C). Furthermore, 1–28 d, 28–56 d, and 56–84 d MCs and WDs all had higher Feed/Body weight gain than Cobb broilers (Figure 1D).

### 3.2. Comparative Analysis of Development in Skeletal Muscle

**Figure 2:**
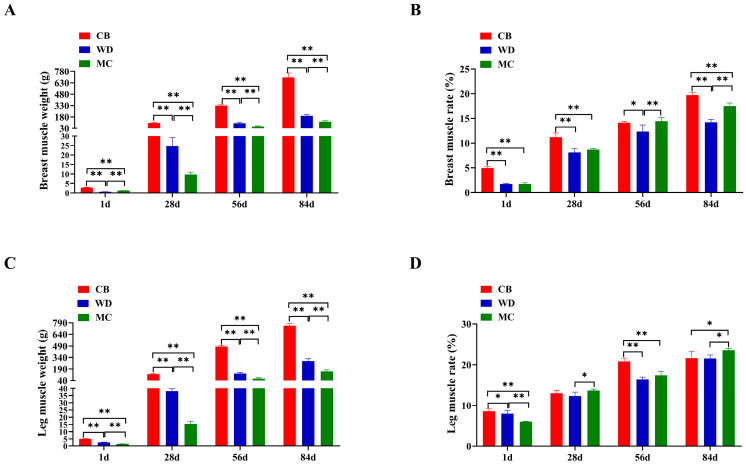
Development in skeletal muscle of three chicken breeds. (**A**) Comparison of breast muscle weight. (**B**) Comparison of breast muscle rate. (**C**) Comparison of leg muscle weight. (**D**) Comparison of leg muscle rate. *: *p* < 0.05; **: *p* < 0.01.

The breast muscle weight followed the order of CB > WD > MC at 28 d, 56 d, and 84 d, but not at 1 d. At 84 d, the breast muscle weight of CBs, WDs, and MCs was 700.01 ± 55.23 g, 194.91 ± 15.93 g, and 116.26 ± 13.29 g, respectively (Figure 2A). The order of breast muscle rate at all ages was CB > MC > WD (Figure 2B). Leg muscle weights at 84 d were 753.78 ± 36.85 g, 292.50 ± 28.26 g, and 157.25 ± 19.15 g for CBs, WDs, and MCs, respectively (Figure 2C). The leg muscle rate was CB, MC > WD at 28 d, 56 d, and 84 d, but not at 1 d (Figure 2D).

### 3.3. Comparative Analysis of Meat Quality Physical Characteristics

Comparisons of the physical parameters of meat quality characteristics of the three breeds were made, including pH, meat color, water loss rate, and shear force (Table 6). The pH 45 min values of the breast and leg muscles of CBs were significantly higher than those of MCs and WDs (*p* < 0.01). The pH 24 h values of the breast muscles of WDs and CBs were significantly lower than those of the leg muscles (*p* < 0.01). The leg muscle color L* values of MCs and CBs were significantly higher than that of WDs (*p* < 0.01). The MC breast muscle color a* value was significantly greater than that of WDs (*p* < 0.05). The breast muscle a* value of WDs was significantly less than that of the leg muscle (*p* < 0.01). The water loss rate of the thigh muscle of CBs was significantly higher than that of WDs (*p* < 0.01). The water loss rate of the breast muscles of CBs was significantly lower than that of the leg muscles (*p* < 0.05). The shearing force of WD breast muscle was higher than that of CBs, while that of CB breast muscle was higher than that of MCs, but the differences were not significant (*p* > 0.05). The shearing force of MC leg muscles was higher than that of WDs (*p* > 0.05). The shearing force of the breast muscles of CBs and WDs was higher than that of the leg muscle (*p* > 0.05).

### 3.4. Comparative Analysis of Meat Quality Chemical Parameters

We then compared the three breeds regarding the chemical composition of the meat, namely, crude ash, CP, CF, water content, and inosine monophosphate content (Table 7). The crude ash contents of the breast muscles of the two indigenous breeds were much higher than that of CBs (*p* < 0.01), and that of MC leg muscles was significantly higher than that of WDs and CBs (*p* < 0.01). When different tissues of the same chicken were compared, the crude ash contents of the breast muscles of the two indigenous breeds were significantly higher than that of the leg muscles (*p* < 0.01). MCs had significantly higher CP in the breast muscle than WDs (*p* < 0.01) and in the leg muscles than CBs (*p* < 0.01). WDs had significantly higher CP in the breast muscles than in the leg muscles (*p* < 0.01). WDs had significantly higher CF in the breast muscle than MCs and CBs (*p* < 0.01). WDs and MCs had significantly higher CF in the leg muscles than CBs (*p* < 0.01). When different tissues of the same breed were compared, MCs, WDs, and CBs all had significantly less CF in the breast muscle than in the leg muscles (*p* < 0.01). CBs had a significantly higher water content in the chest muscles than the other two breeds (*p* < 0.01). WDs had significantly higher water content in the breast muscle than MCs (*p* < 0.05). WDs and CBs had significantly higher water content in the leg muscle than MCs (*p* < 0.01). WDs had significantly higher inosine monophosphate content in the breast muscle than CBs (*p* < 0.01). WDs and MCs had significantly higher inosine monophosphate contents in the leg muscles than CBs (*p* < 0.01).

### 3.5. Comparative Analysis of AA Contents

We then compared the three breeds regarding AA contents (Table 8). WDs had significantly higher contents of essential amino acids (EAAs) and non-essential amino acids (NEAAs) (except cysteine), as well as total amino acids (TAAs), in the breast muscle and thigh muscle than CBs and MCs (*p* < 0.01). CBs and WDs had significantly higher contents of cysteine in the breast muscles than MCs (*p* < 0.05). CBs had lower contents of Thr, Ser, Glu, Gly, Ala, Met, Tyr, Phe, Lys, Arg, and Pro in the breast muscle than in the leg muscle (*p* > 0.05). WDs had lower contents of Thr, Glu, Gly, Ala, Phe, and Pro in the breast muscle than in the leg muscle (*p* > 0.05). MCs had lower contents of Ser, Glu, Cys, Gly, Phe, Arg, and Pro in the breast muscle than in the leg muscle (*p* > 0.05).

### 3.6. Comparative Analysis of FA Contents

We then compared the three breeds for FA contents (Table 9). When comparing different breeds, we used the same tissue. The content of saturated fatty acids (SFAs), monounsaturated fatty acids (MUFAs), polyunsaturated fatty acids (PUFAs), and total fatty acids (TFAs) in the breast muscle of WDs was higher than that of CBs and MCs, and CBs were higher than MCs. WDs had significantly higher contents of C14:1n5, C15:1n5, C16:1n7, C17:1n7, C18:1n9c, and C20:1n in the breast muscle than CBs and MCs (*p* > 0.05). When different tissues of the same breed were compared, CBs had significantly higher contents of SFA, MUFA, PUFA, USFA, EFA, and TFA in the leg muscle than in the breast muscle (*p* < 0.01). WDs had significantly higher contents of C15:0, C17:0, and C20:0 in the leg muscle than in the breast muscle (*p* < 0.05). MCs had significantly higher contents of SFA, MUFA, PUFA, USFA, EFA, and TFA in the leg muscle than in the breast muscle (*p* < 0.05).

### 3.7. Comparative Analysis of Skeletal Muscle Fiber

**Figure 3:**
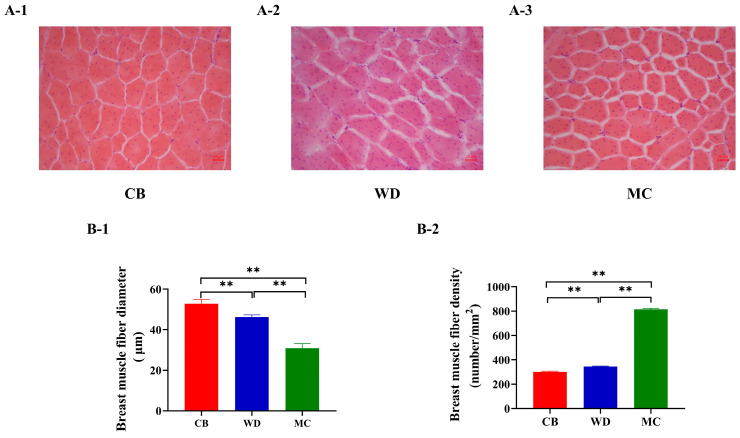
Histological stain of breast muscle fibers of the three chicken breeds. (**A**) Histology of breast muscle fibers at 84 d; H&E stain (10 × 20). (**B**) Comparison of histological characteristics of breast muscle fiber. **: *p* < 0.01.

The breast muscle tissue of the three chicken breeds was analyzed using histological staining. At 84 d, the morphologies of muscle myofibrils in the breast muscles of the three chicken breeds were similar, predominantly elliptical in shape (Figure 3A). The diameter of muscle fibers in CBs was significantly higher than that in WDs and MCs (*p* < 0.01) (Figure 3B-1), and the density of muscle fibers in MCs was significantly higher than that in CBs and WDs (*p* < 0.01) (Figure 3B-2).

**Figure 4:**
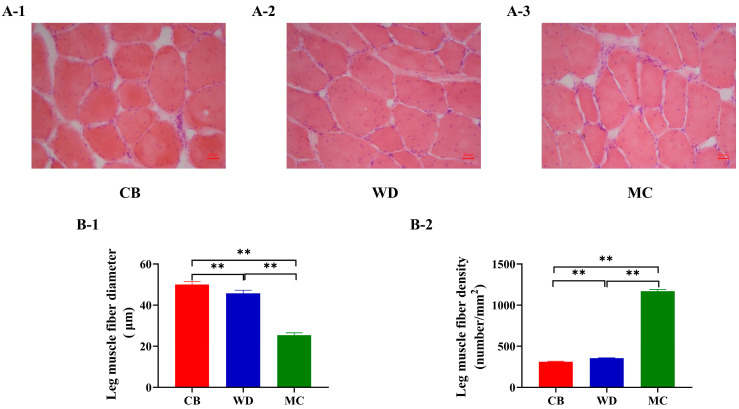
Histological stain of leg muscle fibers of the three chicken breeds. (**A**) Histology of leg muscle fibers in 84 d; H&E stain (10 × 20). (**B**) Comparison of histological characteristics of leg muscle fiber. **: *p* < 0.01.

The leg muscle tissue of the three chicken breeds was analyzed by histological staining. At 84 d, the morphologies of muscle myofibrils in the leg muscles of the three chicken breeds were similar, predominantly elliptical in shape (Figure 4A). The diameter of muscle fibers in CBs was significantly higher than that in WDs and MCs (*p* < 0.01) (Figure 4B-1), and the density of muscle fibers in MCs was significantly higher than that in CBs and WDs (*p* < 0.01) (Figure 4B-2).

### 3.8. Comparative Analysis of RNA-Seq in Breast Muscles

A total of 563315222150 bp paired-end RNA-seq reads were obtained by comparison, with 41 to 54 million reads per sample (Table 10). After aptamer trimming and quality control checks, 562,051,346 clean reads were obtained, all with mass scores above Q30. In total, 68.95 to 82.50% of reads were mapped with the Gallus gallus-5.0 genome (Table 11).

**Figure 5:**
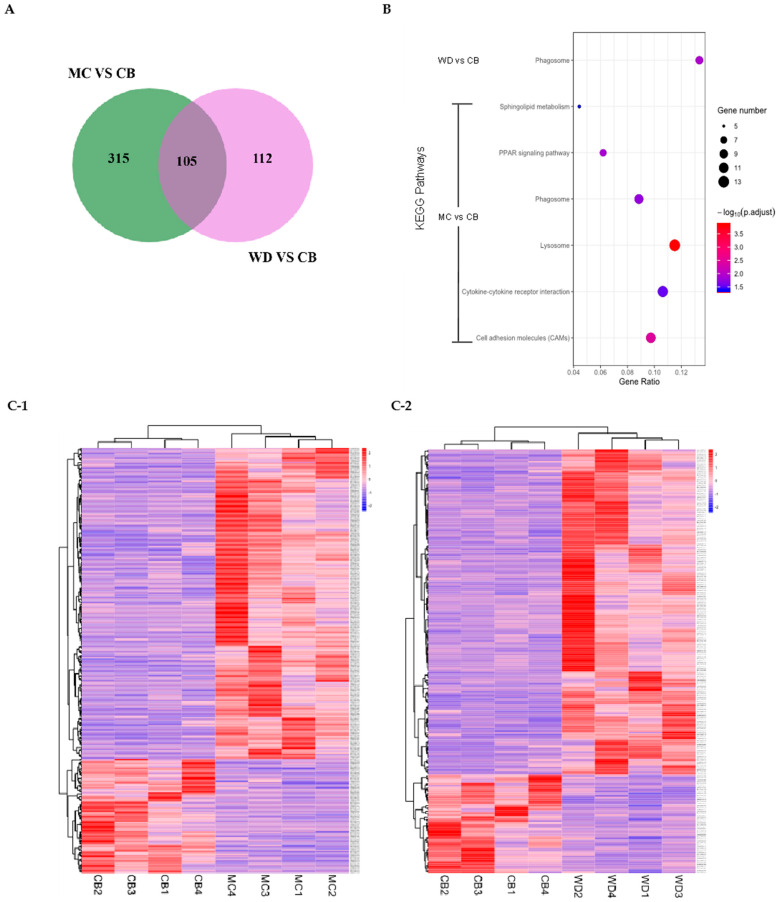
Analysis of DEGs. (**A**) Venn plot of DEGs in MC vs. CB and WD vs. CB groups. Green indicates MC vs. CB. Red indicates WD vs. CB. (**B**) KEGG analysis bubble chart. The *x*-axis represents rich factor (rich factor = number of DEGs enriched in the pathway/number of all genes in the background gene set). The *y*-axis represents the enriched pathway. Color represents enrichment significance, and the size of the bubble represents the number of DEGs enriched in the pathway. (**C**) Heat-map cluster of DEGs in MC vs. CB and WD vs. CB.

The data of MC_vs_CB and WD_vs_CB DEGs were selected for analysis, including 420 DEGs in MC breast muscle relative to CBs and 217 DEGs in WDs relative to CBs. The intersection was taken to draw the Venn diagram (Figure 5A), which yielded a total of 105 shared DEGs.

According to the obtained DEGs, we clustered their expression abundance in each individual from MC vs. CB and WD vs. CB groups. The clustering analysis results showed that only individuals within the same group clustered more closely (Figure 5C).

The bubble chart shows significantly enriched pathways based on DEGs by KEGG pathway analysis *(p* < 0.05). The results show that six pathways were significantly enriched (Figure 5B). Twelve genes (*ATP6V0D2*, *ATP6AP1*, *TCIRG1*, *CTSC*, *CTSV*, *NPC2*, *CTSB*, *HEXA*, *GALC*, *SLC17A5*, *LIPML5*, *LAMP3*, and *SGSH*) were enriched in the lysosome pathway; 11 genes (*CLDN5*, *CLDN1*, *MPZ*, *CLDN19*, *ITGB2*, *ITGB8*, *SIGLEC1*, *CDH2*, *YF5*, *ITGA8*, and *NFASC*) were enriched in the cell adhesion molecules (CAMs) pathway; 7 genes (*PLIN1*, *GK*, *SCD*, *FABP7*, *LPL*, *PPARG*, and *ANGPTL4*) were enriched in the PPAR signaling pathway; the WD vs. CB group had 8 genes (*ATP6V0D2*, *NCF2*, *ATP6V1C2*, *RAB7B*, *TUBA8A*, *CTSV*, *CYBB*, and *TCIRG1*) and the MC vs. CB group had 10 genes (*ATP6V0D2*, *ATP6V1C2*, *ITGB3*, *ATP6AP1*, *TCIRG1*, *NCF2*, *CTSV*, *ITGB2*, *YF5*, and *NCF1*) in the phagosome pathway; 12 genes (*CXCR4*, *CXCL13L3*, *CXCL13L2*, *CCL5*, *GH*, *AMHR2*, *CX3CL1*, *CCR10*, *TNFSF15*, *ACVR1C*, *CXCR1*, and *TNFRSF18*) were enriched in the cytokine–cytokine receptor interaction pathway; and 5 genes (*NEU4*, *GALC*, *UGT8*, *B4GALT6*, and *DEGS2*) were enriched in the sphingolipid metabolism pathway.

**Figure 6:**
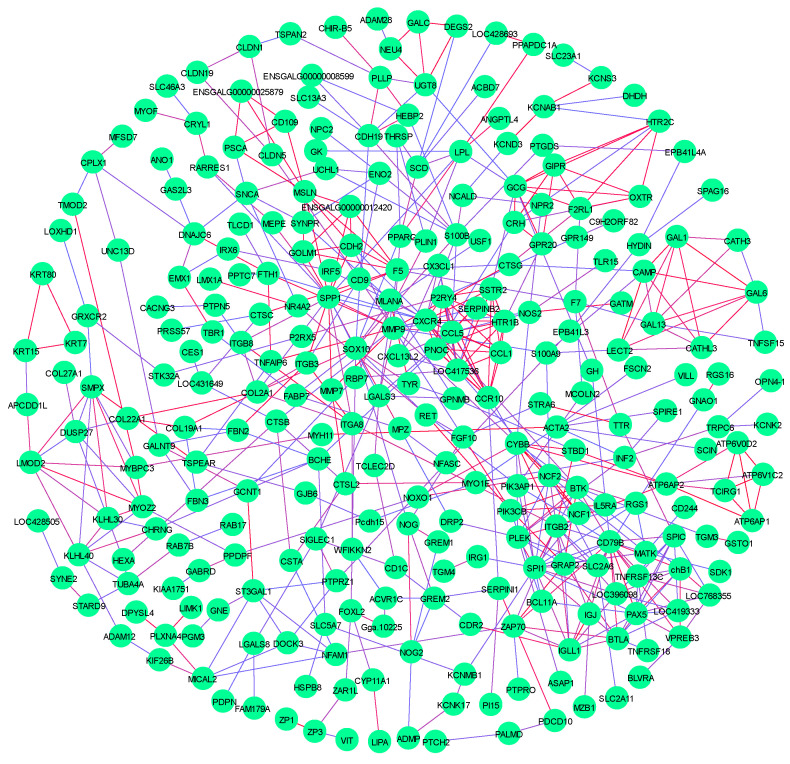
PPI network related to growth performance and meat quality.

The PPI network was constructed by using the extracted target gene list from the STRING database in Cytoscape (Figure 6). The PPI network from DEGs of the MC vs. CB group and the WD vs. CB group comparison contained many protein–protein pairs. Thereinto, IGJ-IGLL1, CDH2-SPP1, PPAPDC1A-LPL, PLIN1-PPARG, SCD-PPARG, MP2-FABP7, and GCG-GH are mainly involved in signal pathways related to growth and meat quality, especially, *LPL*, *PLIN1*, *PPARG*, *SCD*, and *FABP7*. In addition, *IGLL1* and *CDH* are related to immune traits.

### 3.9. qPCR

**Figure 7:**
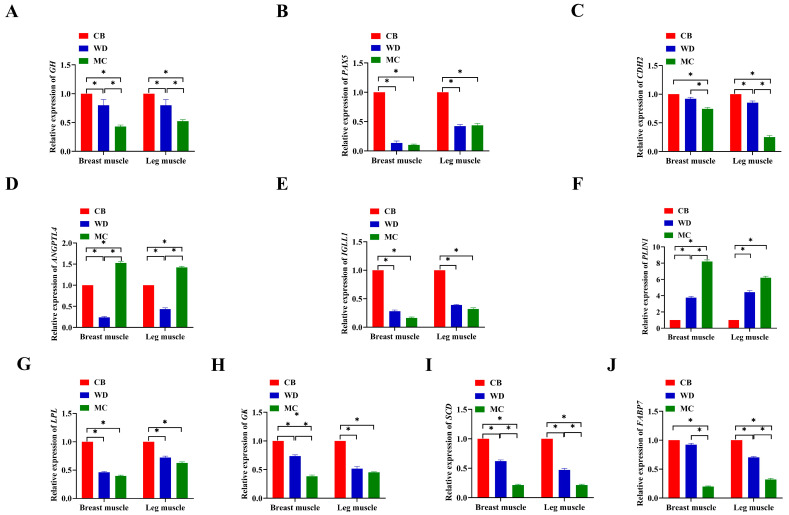
Relative expression levels of 10 genes in three breeds of chicken. *: *p* < 0.05. (**A**) Relative expression of *GH*. (**B**) Relative expression of *PAX5*. (**C**) Relative expression of *CDH2.* (**D**) Relative expression of *ANGPTL4*. (**E**) Relative expression of *IGLL1*. (**F**) Relative expression of *PLIN1*. (**G**) Relative expression of *LPL*. (**H**) Relative expression of *GK*. (**I**) Relative expression of *SCD*. (**J**) Relative expression of *FABP7*.

We examined the expression of 10 genes in the muscles of three breeds of chicken. qPCR was used to detect the relative expression levels of the target genes (Figure 7). The results showed that *GH*, *PAX5*, *CDH2*, *IGLL1*, *LPL*, *GK*, *SCD*, and *FABP7* were highly expressed in CB, moderately expressed in WD, and lowly expressed in MC. *ANGPTL4* and *PLIN1* were highly expressed in MC (*p* < 0.05).

## 4. Discussion

### 4.1. Muscle Meat Quality Characteristics

Since the 20th century, higher meat supply has been a concern of livestock breeding workers, but other indicators related to meat quality have been ignored. For poultry, the combined effects of meat appearance, texture, and flavor are the most important factors in the human diet [2]. Meat is the main end product of poultry, and muscle is one of the major factors, with their weight and quality directly affecting the supply to the market. In this study, the main meat quality characteristics associated with the two indigenous chicken breeds WDs and MCs were compared with those of the highly industrialized CBs. As expected, most indigenous chickens have a unique genetic background that makes their meat quality and flavor unique.

CBs have a significantly higher body weight, daily gain, feed intake, and muscle weight from 1 day to 84 days compared to WDs and MCs (*p* < 0.01). The high intensity of selection and breeding resulted in significant differences in growth performance between CBs and the two indigenous chicken breeds.

The conventional evaluation indicators of chicken quality mainly include pH, shear force, meat color, etc. These physical indicators determine the acceptability of meat products. The pH value can reflect the speed of muscle glycogenolysis after poultry slaughter, and it was an important basis for identifying the quality of meat. After the animal is slaughtered, the glycogen in the body will be broken down into substances such as lactic acid, which will lower the pH of the muscle mass [11]. In this study, the pH of the breast and leg muscles of CBs 45 min after slaughter was higher than in WDs and MCs. When the pH was measured after 24 h, it could be seen that the pH of the breast and leg muscles of CBs, WDs, and MCs decreased. In addition, CBs decreased more. During a 24 h period, the muscles were in the acid excretion stage, which would increase the acidity. Meat color is an important appearance indicator of meat quality, which directly affects consumers’ purchasing desire. The higher the L* value of the meat color, the higher the gloss and the paler the color of the meat. This value was proportional to meat quality, and the b* and L* values are inversely proportional to the meat quality [12]. In this study, the L* and b* values of the breast and leg muscles of CBs and MCs were higher than those of WDs, but the value of the breast and leg muscles of WDs was higher than that of CBs and MCs. It can be seen that the appearance index of the meat quality of WDs was slightly higher than that of CBs and MCs. The water loss rate refers to the ability of muscles to retain water. Muscle water contains many amino acids, vitamins, myoglobin, glycogen, mineral ions, etc. The degree of the water loss rate directly affects the edible quality, including the shape, texture, flavor, texture, and juiciness of the meat [13]. In this study, the water loss rate of the breast and leg muscles of CBs and MCs was higher than in WDs. A high rate of water loss may result in a loss of nutrients contained in muscle water, which can affect the meat quality of the chicken. Shear force can be used to evaluate the tenderness, softness, juiciness, and other characteristics of cooked meat products, which can intuitively reflect the tenderness of chicken. Shear force was significantly affected by the breed of chicken. In a certain range, the smaller the shear force of chicken, the more tender the meat [14]. In the present study, WDs had the highest shear force in the breast muscle, while MCs also had the highest shear force in the leg muscle, but the difference compared to CBs was not significant. Recent studies have shown that meat quality is closely related to muscle fiber traits [15] and the present study showed that the muscle fiber diameter was positively correlated with body weight and muscle rate.

The conventional chemical components of chicken include water, CF, CP, and crude ash. Determining the content of chemical components was one of the ways to judge the quality of meat. The water in the muscle has a great influence on the tenderness, palatability, and juiciness of the meat [16]. CF in the muscle will affect the taste and aroma of the meat. The right amount of fat can ensure the water retention of the meat so that it has a good taste and tenderness. CP content in muscle is the main source of dry matter difference, and crude ash is the oxidized state of mineral elements in muscle [17]. In this study, the contents of CP, CF, and crude ash in the breast and leg muscles of WDs and MCs were higher than those of CBs to varying degrees. The water content of the breast and leg muscles of CBs and WDs was significantly higher than that of MCs. The water content of chicken was generally in the range of 70% to 75%. Within the normal range, the higher the water content, the better the taste. Therefore, the taste of CBs and WDs was slightly better than that of MCs. The higher the fat content within a certain range, the better the quality of the chicken, but if the content is too high, the muscles are easily spoiled. In this study, the fat content of WDs and MCs was higher than that of CBs, so WDs and MCs had better chicken quality. The crude ash content, to some extent, represents the mineral content of the chicken. The higher the crude ash content, the more mineral content or variety in the muscle. Therefore, the mineral content in WDs and MCs was better than that in CBs. As one of the indicators that has attracted much attention in chicken quality research, protein can be hydrolyzed to provide essential amino acids and taste substances needed by the human body [18]. Therefore, WDs have an advantage in terms of protein content.

Inosine monophosphate is an endogenous substance related to meat flavor [19]. Research has shown that the pectoral muscles of five local chicken lines in Korea had higher levels of inosine monophosphate than the leg muscles [20]. In this study, the inosine monophosphate of the breast muscles of these three chicken breeds was not higher than the leg muscles. This result may be caused by different chicken breeds. It was shown by feeding different concentrations of inosine monophosphate that the meat taste of the group with the highest concentration of inosine monophosphate was the best [21]. The inosine monophosphate content in WDs was the highest among the three chicken breeds. This shows that WD meat has a better flavor.

AAs are the basis of major protein synthesis, but they also have other metabolic functions, such as gene expression, synthesis, hormone secretion, nutrient metabolism, oxidative defense, intracellular protein renewal, immune function, reproduction, and lipid metabolism changes [22]. This study found that WDs had the highest EAA and NEAA values. This shows that the AA content of WD muscle was high and has a high edible value. In particular, EAAs provide better sources of AA intake for humans.

Differences in FA types and contents in muscle lead to differences in flavor [23]. The differences in muscle flavor mainly result from fat decomposition and oxidation [24]. Oxide generated by the decomposition of oxidation products continues to generate ketone, aldehyde, acid, and olefine aldehyde compounds, which are also involved in the Maillard reaction. Olefine aldehyde compounds are an especially important precursor of muscles to produce aromatic substances. FAs are one of the hydrolysis products of fats. In this study, SFA, MUFA, PUFA, USFA, EFA, and TFA in WD muscles were higher than those in MCs and CBs. These results suggest that the meat quality of WDs was significantly higher than the other two chicken breeds.

### 4.2. Candidate Genes Associated with Muscle Growth and Meat Quality

*PLIN1*, an important regulator of fat metabolism, plays a role in the formation of lipid droplets by activating the cell death activator *CIDEC* [25]. In addition, *PLIN1* and *LPL* can synergistically regulate lipolysis and triglyceride levels, hydrolyze very low-density lipoproteins, and inhibit the clearance of triglycerides by inhibiting LPL activity. This process involves the recruitment of the proteins PCSK6 and FURIN protease to LPL, leading to the cleavage and dissociation of LP [26]. *FABP7* has been called a brain lipid-binding protein and is a member of the intracellular lipid-binding protein family. This protein can bind and dissolve long-chain PUFAs. It has the characteristics of controlling intracellular lipid dynamics [27]. *PPARG*, PPARG-peroxisome proliferator-activated receptor γ, was a key regulator of adipocyte differentiation and glucose homeostasis. It can bind to peroxisome proliferators. After being activated by ligands, nuclear receptors bind to DNA-specific PPAR response elements (PPREs) and regulate the transcription of their target genes (such as *SCD*) [28]. *SCD* can introduce the first double bond into the saturated fatty acyl-CoA substrate to produce a mixture of C16:1 and C18:1 unsaturated fatty acids [29]. *ANGPTL4* is directly involved in the regulation of glucose homeostasis and lipid metabolism [30]. *GK* is a key member of the FGGY kinase family that regulates glycerol metabolism. GGK can provide G6P during glycolysis [31]. When the body’s free fatty acids and glycerol increase, *GH* can stimulate skeletal muscle lipolysis and lipid oxidation and accelerate the process of fat metabolism [32]. *PAX5* is a B cell-specific activator protein and a redox-sensitive transcription factor. It plays a key role in the entire B lymphocyte production process, being mainly involved in inflammation and immune responses, white blood cell activation, and actin cytoskeleton remodeling. It is also expressed in the brain, B lymphocytes, lymph nodes, and spleen [33]. *IGLL1* belongs to the immunoglobulin gene superfamily. Mutations in *IGLL1* can lead to B-cell defects and globulin hematopathy. If the secretion of immunoglobulin A increases, the expression of *IGLL1* will also increase. The expression of *IGLL1* was closely related to the humoral immune response [34]. *CDH2* plays an important role in adipogenesis, but it was not expressed in mature adipocytes. During myoblast differentiation, it can accelerate the contraction of deposited fibroblast wounds and promote closure. *CDH11* can induce the activation and differentiation of PPARγ receptors and has a certain influence on adipogenesis [35]. The results of this study showed that *GH*, *CDH2*, *PLINI*, *LPL*, *GK*, *SCD*, and *FABK7* were significantly differentially expressed between two native Yunnan chicken breeds and broiler chickens and had a regulatory effect on fat metabolism.

**Figure 8:**
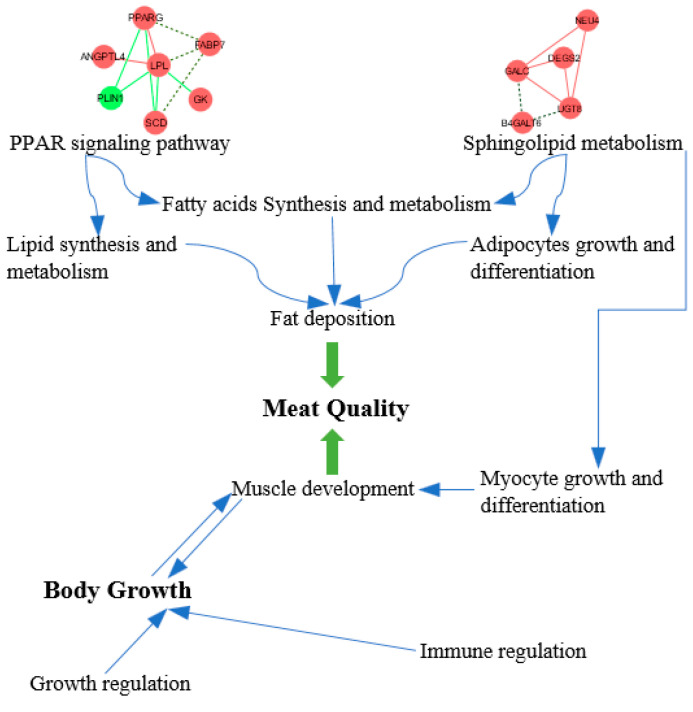
The potential regulatory network of lipid metabolism according to the DEGs enriched in the KEGG pathways.

Based on the identified DEGs, KEGG pathway analysis was carried out to investigate the basic regulatory network underlying differential fat deposition and growth performance in chicken muscle tissue. Among the DEGs associated with lipid metabolism, seven DEGs were significantly enriched in the PPAR signaling pathway (*p* < 0.05), and this pathway is a more classic fat deposition-related pathway [36]. The five DEGs enriched in cell growth and differentiation were significantly enriched in sphingolipid metabolism (*p* < 0.05), it mainly involved in fatty acid synthesis and cell growth and differentiation [37]. Ceramide, the central lipid of sphingolipid biosynthesis, participates in de novo synthesis and proceeds in the Endoplasmic Reticulum. Ceramide is then converted into more complex sphingolipids such as sphingomyelin and glycosphingolipid, which occurs in the Golgi apparatus. Furthermore, the recycling pathway takes place in the lysosome. The lysosomal signaling pathway is mainly involved in the process of cell apoptosis and may interact with the recycling pathway of sphingolipid synthesis [38]. Additionally, DEGs were also significantly enriched (*p* < 0.05) in intracellular and cell interaction signaling pathways (cell adhesion molecules and cytokine–cytokine receptor interactions). In particular, both of these signaling pathways were involved in immune regulation. Cell adhesion molecules were one of the surface membrane molecules of immune cells, which were mainly involved in the recognition function of immune cells, the adhesion between cells, tumor infiltration and metastasis, and intracellular signal transduction. Integrins play important roles in the transport of immune cells to tissues during homeostasis and inflammation, the activation and proliferation of effector cells, and the formation of immune cells [39]. Cytokines are messenger molecules between cells and can bind to the receptors of target cells to produce an immune response. Cytokine receptors can interact with each other, and studies have found that cell surface proteins involved in cell adhesion can support cytokine receptors (GM-CSF/IL-3/IL-5) to enter blood vessels to work, and this was not limited to the inflammatory response [40]. The above research shows that these signaling pathways participate in the immune response. At present, this study has shown that the process of intramuscular fat deposition in chicken breast muscle is also involved in cell junctions, such as tight junctions, extracellular matrix–receptor interactions, focal adhesion, and the regulation of actin cytoskeleton [41]. In addition, a previous study found that the omental, subcutaneous, and intramuscular fat of cattle was enriched in the extracellular receptor interaction [42]. Therefore, it was speculated that immune-related signaling pathways were related to fat deposition, and there were many signaling pathways involved in fat deposition. The specific relationship between them needs to be studied further (Figure 8).

## 5. Conclusions

The two indigenous breeds, WDs and MCs, displayed quite different phenotypes in growth and meat quality compared to the highly industrialized CB breed. These phenotypical differences can be largely explained by the DEGs and the pathways in which they were enriched. Importantly, the results show that four genes in the PPAR signaling pathway related to fat metabolism, namely *LPL*, *GK*, *SCD*, and *FABP7*, were differentially expressed between native breeds and broilers. This study revealed that the genes in this study could be used as molecular screening markers in future molecular breeding for chicken meat quality traits to reveal the mechanisms of fat deposition in poultry at the molecular level.

## Figures and Tables

**Table 1:** Diet composition and nutrition level.

Compositions of Diets %	Phase I	Phase II
Corn	58.85	61.25
Soy protein	25.29	22.44
Wheat bran	8.90	9.50
Fish meal	3.00	3.00
Calcium hydrogen phosphate	1.47	1.41
Limestone	1.10	1.00
Lys	0	0.02
Met	0.12	0.11
Sodium Chloride	0.27	0.27
Minerals and vitamins	1.00	1.00
Total	100	100
Nutrients levels		
Metabolism energy (MJ·kg^−1^)	12.00	12.10
Crude protein	18.50	17.00
Calcium	0.95	0.95
Phosphorus	0.68	0.65
Lys	0.96	0.95
Met	0.40	0.38

Note: Main composition of premix (converted to ration per kg): VA 15,000 U, VD 33,300 U, VE 62.5 mg, VK 3.6 mg, VBl 3 mg, VB2 9 mg, VB6 6 mg, VB12 0.03 mg, Nicotinamide 60 mg, D-pantothenic acid 18 mg, Folic acid 1.5 mg, Biotin 0.36 mg, Choline chloride 600 mg, Fe 80 mg, Cu 12 mg, Zn 75 mg, Mn 60 mg, I 0.35 mg, Se 0.15 mg, and antibacterial growth promoters, antioxidants.

**Table 2:** Vaccination protocol.

Age (Day)	Vaccine	Way
1	Marek’s disease	Subcutaneous injection
3	Newcastle	Oral vaccination
12	Gumboro	Subcutaneous injection
20	Newcastle	Oral vaccination
42	Fowl cholera	Subcutaneous injection

**Table 3:** Feeding management standards.

Age (Day)	Density (Birds/m^2^)	Temperature (°C)	Relative Humidity (%)	Light Intensity
1–3	15	33–35	65–70	25 Lx, 24 h
4–7	15	30–33	65–70	10 Lx, 23 h
8–14	15	28–30	60–65	10 Lx, 23 h
15–21	6	26–28	55–60	8 Lx, 18 h
22–28	6	24–26	55–60	8 Lx, 18 h
29–35	3	21–24	55	8 Lx, 18 h
36–84	3	18–21	55	5 Lx, 18 h

**Table 4:** Muscle development evaluation measurement methods.

Items	Measurement Methods
Breast muscle weight	The weight of the breast without skin and adherent fat
Breast muscle rate	Percentage of breast muscle weight in body weight
Leg muscle weight	The weight of the two legs without skin and adherent fat
Leg muscle rate	Percentage of leg muscle weight in body weight

**Table 5:** Primers for 10 genes for qPCR validation.

Gene	Primer Sequence (5′-3′)	Annealing Temperature (°C)	Product Size (bp)
*β-actin*	F: tggactcctacaaccaacgg R: catcctccttgaactcgcag	58.8	258
*PLIN1*	F: atggtgagaggcagagcatt R: cttcttcacgctggagatgc	56.6	185
*LPL*	F: ggttcctggacagatggaca R: caacatcctttcccaccagc	58.8	239
*FABP7*	F: tgacgaatacatgaaggcgc R: catcaaattcctcgccgagt	58.3	167
*SCD*	F: caagttctccgagacgcatg R: gggcttgtagtatctccgct	56.6	178
*ANGPTL4*	F: tggaagactgggagggaaac R: gtttgtgtccgctttgaggt	57.6	185
*GK*	F: cgggaacttcttatggctgc R: aatggtatcccgcagtcctt	58.3	202
*PAX5*	F: atcagcaagtcccagtctcc R: gtctccacgcatctgtttcc	57.6	239
*IGLL1*	F: accaacagaccctcgaacat R: ttgtcccggccccaaatata	58.8	157
*CDH2*	F: caaaactttcggaccctgca R: gtggtggcttcttttgggtt	58.8	232
*GH*	F: agctgcttcggttttcactg R: atcgtaggtgggtctgagga	57.6	209

**Table 6:** Meat quality physical indexes of three chicken breeds.

Items	CB		WD		MC	
	Breast Muscle	Leg Muscle	Breast Muscle	Leg Muscle	Breast Muscle	Leg Muscle
pH 45 min	6.40 ± 0.37 ^A^	6.55 ± 0.17 ^A^	5.95 ± 0.12 ^B^	6.41 ± 0.26 ^B^	5.89 ± 0.26 ^B^	6.17 ± 0.10 ^B^
pH 24 h	5.92 ± 0.35	6.55 ± 0.22 **	5.69 ± 0.21	6.40 ± 0.62 **	5.66 ± 0.10	6.09 ± 0.15
L*	50.16 ± 4.21	52.04 ± 3.63 ^A^	46.91 ± 3.78	42.22 ± 2.8 ^B^	50.82 ± 2.06	50.43 ± 2.26 ^A^
a*	3.47 ± 2.29 ^c^	7.64 ± 2.46 ^Bb^*	4.26 ± 1.46 ^b^	14.27 ± 4.79 ^Aa^**	5.65 ± 1.08 ^a^	9.36 ± 2.68 ^b^
b*	6.37 ± 1.67	5.97 ± 3.20	6.13 ± 3.12	5.35 ± 1.73	6.26 ± 1.79	5.58 ± 0.80
water loss rate (%)	13.49 ± 2.62	19.34 ± 4.54 ^A^*	9.77 ± 1.75	9.38 ± 2.01 ^B^	11.52 ± 3.74	11.34 ± 2.91 ^AB^
shearing force (kg/f)	4.71 ± 1.32	3.70 ± 2.52	4.73 ± 1.46	4.37 ± 1.19	4.46 ± 1.02	5.21 ± 0.97

Note: Asterisk indicates that the differences in muscles from different parts of the same chicken breed are significant (*: *p* < 0.05; **: *p* < 0.01). The letters indicate that the differences in the muscles from the same parts of different chicken breeds are significant (lower case letter: *p* < 0.05; upper case letters: *p* < 0.01).

**Table 7:** Meat quality chemical parameters of three chicken breeds.

Items	CB		WD		MC	
	Breast Muscle	Leg Muscle	Breast Muscle	Leg Muscle	Breast Muscle	Leg Muscle
Crude ash (%)	1.33 ± 0.09 ^B^	1.27 ± 0.02 ^B^	1.72 ± 0.14 ^A^**	1.35 ± 0.03 ^B^	1.72 ± 0.03 ^A^**	1.59 ± 0.07 ^A^
CP (%)	24.26 ± 0.40 ^C^	24.46 ± 0.57 ^B^	27.30 ± 0.52 ^B^**	23.68 ± 0.18 ^C^	28.37 ± 0.51 ^A^	28.23 ± 0.13 ^A^
CF (%)	0.46 ± 0.05 ^C^	0.85 ± 0.20 ^B^**	0.86 ± 0.051 ^A^	2.08 ± 0.36 ^A^**	0.67 ± 0.02 ^B^	1.90 ± 0.05 ^A^**
Water (%)	73.95 ± 0.45 ^A^	73.43 ± 0.77 ^A^	70.12 ± 0.70 ^Ba^	72.89 ± 0.54 ^A^	69.23 ± 0.53 ^Bb^	68.29 ± 0.12 ^B^
Inosine monophosphate (%)	1.01 ± 0.15 ^Bb^	1.04 ± 0.14 ^B^	1.28 ± 0.11 ^A^	1.35 ± 0.04 ^A^	1.23 ± 0.09 ^a^	1.30 ± 0.07 ^A^

Note: Asterisk indicates that the differences in muscles from different parts of the same chicken breed are significant (**: *p* < 0.01). The letters indicate that the differences in the muscles from the same parts of different chicken breeds are significant (lower case letter: *p* < 0.05; upper case letters: *p* < 0.01).

**Table 8:** Amino acid contents of the three chicken breeds (air-dry sample).

Items		CB		WD		MC	
		Breast Muscle	Leg Muscle	Breast Muscle	Leg Muscle	Breast Muscle	Leg Muscle
**EAA**	Thr	2.81 ± 0.23 ^B^	2.83 ± 0.12 ^B^	4.25 ± 0.12 ^A^	4.26 ± 0.25 ^A^	2.64 ± 0.10 ^B^	2.63 ± 0.15 ^B^
	Val	3.11 ± 0.18 ^B^	2.98 ± 0.17 ^B^	4.78 ± 0.07 ^A^	4.45 ± 0.24 ^A^	2.97 ± 0.16 ^B^	2.80 ± 0.13 ^B^
	Met	1.53 ± 0.04 ^B^	1.57 ± 0.10 ^B^	2.37 ± 0.13 ^A^	2.37 ± 0.12 ^A^	1.50 ± 0.07 ^B^	1.46 ± 0.11 ^B^
	Ile	2.99 ± 0.20 ^B^	2.94 ± 0.17 ^B^	4.41 ± 0.11 ^A^	4.29 ± 0.23 ^A^	2.79 ± 0.15 ^B^	2.75 ± 0.09 ^B^
	Leu	4.94 ± 0.32 ^B^	4.91 ± 0.24 ^B^	7.54 ± 0.20 ^A^	7.42 ± 0.37 ^A^	4.68 ± 0.20 ^B^	4.60 ± 0.20 ^B^
	Phe	1.56 ± 0.11 ^B^	1.68 ± 0.10 ^B^	2.89 ± 0.52 ^A^	3.08 ± 0.60 ^A^	1.51 ± 0.06 ^B^	1.59 ± 0.09 ^B^
	Lys	5.50 ± 0.33 ^B^	5.53 ± 0.26 ^B^	8.27 ± 0.24 ^A^	8.18 ± 0.52 ^A^	5.16 ± 0.16 ^B^	5.05 ± 0.32 ^B^
**NEAA**	Asp	5.80 ± 0.43 ^B^	5.77 ± 0.32 ^B^	8.59 ± 0.24 ^A^	8.34 ± 0.46 ^A^	5.48 ± 0.30 ^B^	5.38 ± 0.27 ^B^
	Ser	2.39 ± 0.18 ^B^	2.52 ± 0.11 ^B^	3.70 ± 0.17 ^A^	3.67 ± 0.26 ^A^	2.29 ± 0.06 ^B^	2.38 ± 0.15 ^B^
	Glu	9.32 ± 0.65 ^B^	9.83 ± 0.83 ^B^	13.82 ± 0.56 ^A^	14.52 ± 0.76 ^A^	8.57 ± 0.39 ^B^	8.88 ± 0.42 ^B^
	Gly	2.92 ± 0.33 ^B^	3.24 ± 0.48 ^B^	4.13 ± 0.23 ^A^	4.61 ± 0.39 ^A^	2.81 ± 0.30 ^B^	3.24 ± 0.32 ^B^
	Ala	3.79 ± 0.28 ^B^	3.82 ± 0.33 ^B^	5.32 ± 0.49 ^A^	5.52 ± 0.42 ^A^	3.57 ± 0.17 ^B^	3.54 ± 0.18 ^B^
	Cys	0.62 ± 0.06 ^a^	0.42 ± 0.03	0.55 ± 0.07 ^a^	0.39 ± 0.10	0.46 ± 0.10 ^b^	0.59 ± 0.15
	Tyr	1.78 ± 0.13 ^B^	1.79 ± 0.09 ^B^	2.98 ± 0.22 ^A^	2.84 ± 0.14 ^A^	1.67 ± 0.08 ^B^	1.65 ± 0.10 ^B^
	His	2.48 ± 0.34 ^B^	2.24 ± 0.48 ^B^	4.24 ± 0.13 ^A^	3.98 ± 0.31 ^A^	2.32 ± 0.32 ^B^	2.17 ± 0.25 ^B^
	Arg	3.78 ± 0.26 ^B^	3.86 ± 0.26 ^B^	6.29 ± 0.18 ^A^	6.25 ± 0.32 ^A^	3.63 ± 0.20 ^B^	3.64 ± 0.18 ^B^
	Pro	1.13 ± 0.14 ^C^	1.74 ± 0.35 ^B^	2.66 ± 0.04 ^A^	3.02 ± 0.22 ^A^	1.50 ± 0.14 ^B^	1.71 ± 0.11 ^B^
**TAA**		56.43 ± 2.13 ^B^	57.66 ± 2.20 ^B^	86.78 ± 3.10 ^A^	87.28 ± 3.20 ^A^	53.54 ± 1.95 ^B^	54.06 ± 1.97 ^B^

Note: The letters indicate that the differences in the muscles from the same parts of different chicken breeds are significant (lower case letter: *p* < 0.05; upper case letters: *p* < 0.01).

**Table 9:** Fatty acid contents of the three chicken breeds (lyophilized samples).

Items	CB		WD		MC	
	Breast Muscle	Leg Muscle	Breast Muscle	Leg Muscle	Breast Muscle	Leg Muscle
C12:0	0.018 ± 0.003 ^b^	0.050 ± 0.005 ^a^*	0.025 ± 0.003 ^a^	0.028 ± 0.010 ^b^	0.000 ± 0.000 ^c^	0.000 ± 0.000 ^c^
C14:0	0.305 ± 0.027 ^B^	1.285 ± 0.13 ^A^**	0.665 ± 0.077 ^A^	0.875 ± 0.321 ^B^	0.125 ± 0.014 ^C^	0.243 ± 0.015 ^C^*
C15:0	0.040 ± 0.004 ^b^	0.183 ± 0.018 ^a^**	0.068 ± 0.008 ^a^	0.133 ± 0.049 ^a^*	0.018 ± 0.002 ^c^	0.040 ± 0.002 ^b^*
C16:0	15.088 ± 1.355 ^B^	50.978 ± 5.165 ^A^**	24.720 ± 2.869 ^A^	31.813 ± 11.67 ^B^	6.375 ± 0.712 ^C^	10.950 ± 0.684 ^C^*
C17:0	0.063 ± 0.006 ^b^	0.323 ± 0.033 ^a^**	0.128 ± 0.015 ^a^	0.265 ± 0.097 ^a^*	0.060 ± 0.007 ^b^	0.120 ± 0.007 ^b^*
C18:0	7.210 ± 0.648 ^B^	18.308 ± 1.855 ^A^**	9.393 ± 1.09 ^A^	11.758 ± 4.316 ^B^	3.490 ± 0.39 ^C^	7.685 ± 0.48 ^B^*
C20:0	0.060 ± 0.005 ^b^	0.260 ± 0.026 ^a^**	0.115 ± 0.013 ^a^	0.443 ± 0.162 ^a^*	0.025 ± 0.003 ^c^	0.070 ± 0.004 ^b^*
C22:0	0.073 ± 0.007 ^a^	0.078 ± 0.008	0.040 ± 0.005 ^b^	0.058 ± 0.021	0.028 ± 0.003 ^c^	0.058 ± 0.004 *
C24:0	0.545 ± 0.049 ^a^	0.690 ± 0.07 *	0.480 ± 0.056 ^a^	0.573 ± 0.21	0.333 ± 0.037 ^b^	0.510 ± 0.032 *
C14:1n5	0.095 ± 0.009 ^b^	0.493 ± 0.05 ^A^**	0.130 ± 0.015 ^a^	0.220 ± 0.081 ^B^	0.015 ± 0.002 ^c^	0.055 ± 0.003 ^C^*
C15:1n5	0.118 ± 0.011 ^a^*	0.053 ± 0.005 ^a^	0.128 ± 0.015 ^a^**	0.020 ± 0.007 ^b^	0.000 ± 0.000 ^b^	0.010 ± 0.001 ^b^*
C16:1n7	3.018 ± 0.271 ^B^*	1.598 ± 0.162 ^B^	5.078 ± 0.589 ^A^	7.273 ± 2.669 ^A^	0.750 ± 0.084 ^C^	1.845 ± 0.115 ^B^*
C17:1n7	0.065 ± 0.006 ^b^	0.185 ± 0.019 ^a^*	0.155 ± 0.018 ^a^	0.190 ± 0.07 ^a^	0.018 ± 0.002 ^c^	0.058 ± 0.004 ^b^*
C18:1n9c	17.918 ± 1.609 ^B^	72.878 ± 7.384 ^A^**	39.915 ± 4.632 ^A^	51.053 ± 18.73 ^A^	9.583 ± 1.07 ^C^	16.600 ± 1.037 ^B^*
C20:1n	0.220 ± 0.020 ^b^	1.118 ± 0.113 ^a^**	0.823 ± 0.095 ^a^	1.065 ± 0.391 ^a^	0.158 ± 0.018 ^b^	0.320 ± 0.02 ^b^*
C18:2n6c	10.498 ± 0.943 ^B^	35.703 ± 3.618 ^A^**	20.665 ± 2.398 ^A^	30.825 ± 11.310 ^A^	6.69 ± 0.747 ^C^	12.983 ± 0.811 ^B^*
C18:3n6	0.093 ± 0.008 ^b^	0.383 ± 0.039 ^a^**	0.143 ± 0.017 ^a^	0.253 ± 0.093 ^b^	0.088 ± 0.01 ^b^	0.165 ± 0.01 ^b^*
C18:3n3	0.298 ± 0.027 ^b^	1.360 ± 0.138 ^a^**	0.645 ± 0.075 ^a^	1.058 ± 0.388 ^a^	0.220 ± 0.025 ^b^	0.413 ± 0.026 ^b^*
C20:2n6	0.185 ± 0.017 ^a^	0.410 ± 0.042 ^a^*	0.223 ± 0.026 ^a^	0.408 ± 0.15 ^a^	0.123 ± 0.014 ^b^	0.233 ± 0.015 ^b^*
C20:4n6	5.638 ± 0.506 ^a^	7.925 ± 0.803 ^A^*	4.350 ± 0.505 ^b^	5.413 ± 1.987 ^B^	3.140 ± 0.351 ^c^	5.530 ± 0.346 ^B^*
C20:5n3	0.070 ± 0.006	0.125 ± 0.013	0.073 ± 0.008	0.115 ± 0.042	0.075 ± 0.008	0.083 ± 0.005
C24:6n3	0.598 ± 0.054 ^a^	0.778 ± 0.079 ^a^*	0.398 ± 0.046 ^b^	0.538 ± 0.197 ^b^	0.390 ± 0.044 ^b^	0.773 ± 0.048 ^a^*
SFA	23.400 ± 2.102 ^B^	72.153 ± 7.311 ^A^**	35.633 ± 4.135 ^A^	45.943 ± 16.86 ^B^	10.453 ± 1.167 ^C^	19.675 ± 1.23 ^C^*
MUFA	21.433 ± 1.925 ^B^	76.323 ± 7.733 ^A^**	46.228 ± 5.365 ^A^	59.820 ± 21.957 ^A^	10.523 ± 1.175 ^C^	18.888 ± 1.18 ^B^*
PUFA	17.378 ± 1.561 ^B^	46.683 ± 4.73 ^A^**	26.495 ± 3.075 ^A^	38.608 ± 14.17 ^A^	10.725 ± 1.197 ^C^	20.178 ± 1.261 ^B^*
USFA	38.810 ± 3.486 ^B^	123.005 ± 12.46 ^A^**	72.723 ± 8.439 ^A^	98.428 ± 36.12 ^A^	21.248 ± 2.372 ^C^	39.065 ± 2.441 ^B^*
EFA	16.525 ± 1.484 ^b^	45.370 ± 4.597 ^A^**	25.803 ± 2.994 ^A^	37.548 ± 13.78 ^A^	10.138 ± 1.132 ^c^	19.090 ± 1.193 ^B^*
TFA	62.210 ± 5.587 ^B^	195.158 ± 19.77 ^A^**	108.355 ± 12.57 ^A^	144.370 ± 52.99 ^A^	31.700 ± 3.539 ^C^	58.740 ± 3.671 ^B^*

Note: Asterisk indicates that the differences in muscles from different parts of the same chicken breed are significant (*: *p* < 0.05; **: *p* < 0.01). The letters indicate that the differences in the muscles from the same parts of different chicken breeds are significant (lower case letter: *p* < 0.05; upper case letters: *p* < 0.01).

**Table 10:** Summary of the RNA-seq data.

Sample		Raw Data		Clean Data		Clean Data Ratio	
		Sequences	Bases	Sequences	Bases	Sequences	Bases
CB1	read1	44157511	6554832118	43990235	6444213183	99.62%	98.31%
	read2		6554832118		6445842898		98.34%
CB2	read1	48373644	7130323676	48212083	7021232185	99.67%	98.47%
	read2		7130323676		7024719635		98.52%
CB3	read1	50417672	7455132971	50360438	7407469730	99.89%	99.36%
	read2		7455132971		7407611791		99.36%
CB4	read1	50343815	7448999742	50260894	7382587236	99.84%	99.11%
	read2		7448999742		7386118122		99.16%
CB5	read1	47428679	7021075183	47242571	6906712188	99.61%	98.37%
	read2		7021075183		6904694579		98.34%
WD1	read1	41193762	6095723172	41151375	6058668197	99.90%	99.39%
	read2		6095723172		6058855779		99.40%
WD2	read1	46889983	6973814175	46789831	6906779152	99.79%	99.04%
	read2		6973814175		6909960586		99.08%
WD3	read1	54172288	8043390125	54046615	7963234169	99.77%	99.00%
	read2		8043390125		7966400727		99.04%
MC1	read1	43635413	6496325561	43576854	6455586208	99.87%	99.37%
	read2		6496325561		6453106964		99.33%
MC2	read1	46462679	6897145314	46389506	6836428320	99.84%	99.12%
	read2		6897145314		6842573604		99.21%
MC3	read1	45892152	6813870731	45820588	6758336835	99.84%	99.18%
	read2		6813870731		6760233491		99.21%
MC4	read1	44347624	6564043642	44210356	6469829352	99.69%	98.56%
	read2		6564043642		6470469881		98.57%

Note: Raw data: Raw data volume statistics, sequencing sequence number, and total number of bases for each sample; Clean data: The effective data volume statistics, the number of reads, and the total number of bases after mass pretreatment of each sample; Clean data Ratio: valid data ratio, (clean reads/raw reads) × 100.

**Table 11:** Statistical analysis of mapping data.

Sample	Clean Reads	Mapped Reads	Mapped Ratio
CB1	43990235	33107050	75.26%
CB2	48212083	36578507	75.87%
CB3	50360438	39402006	78.24%
CB4	50260894	37509705	74.63%
WD1	47242571	34699668	73.45%
WD2	41151375	33949884	82.50%
WD3	46789831	35087694	74.99%
WD4	54046615	39697238	73.45%
MC1	43576854	32656494	74.94%
MC2	46389506	36346177	78.35%
MC3	45820588	34837393	76.03%
MC4	44210356	30483040	68.95%

Note: Clean reads: Number of reads of each sample after quality control preprocessing; Compare the number of valid reads on the reference genome; Mapped ratio: (Mapped Reads/Clean Reads) × 100.

## Data Availability

The original contributions presented in the study are included in the article, further inquiries can be directed to the corresponding author.
